# Comparison of MRI and High-Resolution USG in Evaluating Patients With Rotator Cuff Pathologies

**DOI:** 10.7759/cureus.72497

**Published:** 2024-10-27

**Authors:** Suchithra S Soundararajan, Shaik Farid

**Affiliations:** 1 Radiology, Karpaga Vinayaga Institute of Medical Sciences and Research Centre, Chennai, IND; 2 Radiodiagnosis, Karpaga Vinayaga Institute of Medical Sciences and Research Centre, Chennai, IND

**Keywords:** diagnostic accuracy, magnetic resonance imaging, rotator cuff, shoulder pain, ultrasonography

## Abstract

Background

Accurate diagnosis of rotator cuff pathologies is crucial for the effective management of shoulder pain. This study aimed to evaluate the diagnostic accuracy of high-resolution USG and MRI for detecting rotator cuff injuries.

Methods

A retrospective study was conducted on 40 patients with shoulder pain who underwent both USG and MRI over six months at the Department of Radiodiagnosis, Karpaga Vinayaga Institute of Medical Sciences and Research Centre in Chennai, India, in collaboration with the Department of Orthopedics. The diagnostic accuracy metrics of USG were compared to those of MRI, considered the reference standard, and analyzed using IBM SPSS Statistics for Windows, Version 28.0 (Released 2021; IBM Corp., Armonk, NY, USA).

Results

The mean age of the 40 patients was 46.12 ± 15.31 years. Among them, males represented the majority (n = 27, 67.5%), with a significant number having diabetes mellitus (n = 14, 35%) and hypertension (n = 9, 22.5%). The right shoulder was the most frequently affected (n = 33, 82.5%). USG identified supraspinatus tears in 33 patients (82.5%), subscapularis tears in 16 patients (40.0%), and infraspinatus tears in two patients (5.0%). MRI detected supraspinatus tears in 39 patients (97.5%), subscapularis tears in 18 patients (45.0%), and infraspinatus tears in two patients (5.0%). The diagnostic performance of USG demonstrated a sensitivity of 76.92% and specificity of 85.71%, while MRI exhibited a sensitivity of 92.86% and specificity of 80.77%.

Conclusions

Both USG and MRI are valuable for diagnosing rotator cuff pathologies, and MRI provides superior sensitivity and specificity. However, USG remains a reliable and cost-effective initial diagnostic tool, particularly when used in conjunction with MRI for comprehensive assessment.

## Introduction

The shoulder joint is a highly mobile and complex structure, characterized by its incongruous large ball and small socket design, which allows extensive motion across multiple planes, but often at the expense of stability. The muscles and tendons associated with this joint are frequently subjected to significant strain, leading to substantial wear and tear, especially during athletic activities. The underlying causes of shoulder pain can vary widely from acute trauma to chronic degenerative conditions, including impingement syndrome. Although numerous clinical tests have been developed to diagnose shoulder pain and are generally effective in identifying periarticular lesions, accurately identifying shoulder pathology through physical examination alone remains challenging. Clinical diagnoses are often less accurate than arthroscopic findings [1,2].

Rotator cuff injuries encompass a spectrum of chronic conditions that primarily affect the musculotendinous junction, osteotendinous components, and anatomically restricted subacromial space. Consequently, ultrasound has become a widely used imaging modality for evaluating these injuries. Advances in ultrasound technology, including improved resolution and refined techniques. A better understanding of shoulder pathologies has significantly enhanced the diagnostic accuracy for rotator cuff disorders [3]. High-resolution ultrasound offers a noninvasive, cost-effective, and non-ionizing alternative with considerable sensitivity for detecting both rotator cuff and non-rotator cuff abnormalities [4].

Although plain radiography remains a fundamental initial tool for assessing bony trauma and various arthropathies, it is often necessary to supplement it with additional imaging techniques, such as arthrography, or modalities specifically designed to evaluate soft tissue structures. Both MRI and USG have largely replaced arthrography in assessing rotator cuff integrity. MRI, in particular, has become the “gold standard” for detecting both subtle and overt internal derangements and for providing comprehensive assessments of joint structures. Its multiplanar imaging capability and superior soft tissue contrast make it an invaluable tool for the evaluation of shoulder pathology [5,6].

Over the past two decades, musculoskeletal USG has emerged as a versatile imaging modality, particularly in sports medicine and rheumatology. It has gained significant recognition in the literature along with MRI. The real-time imaging capability of ultrasound, which allows for dynamic studies of the shoulder, is especially advantageous because it enables patient interaction and identification of pain or discomfort associated with specific movements or positions [7,8].

A limited number of studies have assessed high-resolution USG and MRI findings for evaluating the injury of rotator cuff pathologies in patients presenting with shoulder pain. Our study aimed to cover a gap in Indian participants who were diagnosed with chronic shoulder pain and could have a potential rotator cuff injury.

This study aimed to assess the diagnostic accuracy of high-resolution USG and MRI for the evaluation of rotator cuff pathologies in patients presenting with shoulder pain.

## Materials and methods

Study design and population

This retrospective study was conducted on patients with shoulder pain who were referred for high-resolution USG and MRI over six months (October 2023 to March 2024) in the Department of Radiodiagnosis, in collaboration with the Department of Orthopedics at the Karpaga Vinayaga Institute of Medical Sciences and Research Centre, Chennai, India.

Sample size estimation

In a study conducted by Minagawa et al., the prevalence of rotator cuff injuries was 22.1% [9]. Using this parameter, the sample size was estimated and calculated as (n = 40).

Data collection procedures

The principal investigator collected the primary data. Clinical examination findings, ultrasound examination of the shoulder, and MRI findings were collected. The ultrasound and MRI findings were correlated and tabulated for further analysis.

Data management and statistical analysis

Data were entered into Microsoft Excel (Microsoft Corporation, Redmond, WA, USA) and analyzed using IBM SPSS Statistics for Windows, Version 28.0 (Released 2021; IBM Corp., Armonk, NY, USA). Continuous variables are presented as mean and SD, while categorical variables are presented as percentages. An unpaired t-test was used to compare the means between groups. Associations between categorical variables were determined using the chi-squared test or Fisher’s exact test, as appropriate. Statistical significance was set at p < 0.05. The sensitivity, specificity, accuracy, positive predictive value (PPV), and negative predictive value (NPV) of ultrasound compared with MRI were calculated for each parameter.

Ethical considerations

Ethical clearance was obtained before the commencement of the study (approval number KIMS/PG/03/08.05.2024).

Implications of the study

MRI was found to be a superior and more sensitive tool than ultrasound for detecting and grading musculoskeletal injuries, particularly in cases of partial tears and tendinopathy. However, ultrasound proved to be a reliable initial evaluation tool for rotator cuff injuries, with an accuracy comparable to that of MRI. MRI provides a better anatomical delineation of rotator cuff injuries, which is critical for surgical planning.

## Results

Our study included a total of 40 patients with a mean age of 46.12 ± 15.31 years. A male predominance was observed, with 27 males (67.5%) and 13 females (32.5%) enrolled. A significant proportion of patients had diabetes mellitus (n = 14, 35%), while hypertension was reported in nine patients (22.5%). The right shoulder was more frequently affected, with 33 cases (82.5%) involving the right side, compared to seven cases (17.5%) involving the left shoulder (Table 1).

**Table 1:** Patient baseline and rotator cuff characteristics

Characteristics (N = 40)		N	%
Gender distribution	Males	27	67.5
	Females	13	32.5
Comorbidities	Diabetes mellitus	14	35
	Hypertension	9	22.5
Side of involvement	Right	33	82.5
	Left	7	17.5
USG rotator cuff tears	Supraspinatus	33	82.5
	Subscapularis	16	40
	Infraspinatus	2	5
Pathologies of rotator cuff on USG	Partial tear	22	55
	Complete tear	18	45
MRI rotator cuff tears	Supraspinatus	39	97.5
	Subscapularis	18	45
	Infraspinatus	2	5
Pathologies of rotator cuff on MRI	Partial tear	26	65
	Complete tear	14	35

USG findings

USG identified supraspinatus tears in 82.5% (n = 33), subscapularis tears in 40.0% (n = 16), and infraspinatus tears in 5.0% (n = 2) of patients. Regarding the extent of rotator cuff pathology, USG detected partial tears in 55.0% (n = 22) of the patients and complete tears in 45.0% (n = 18). MRI revealed a higher prevalence of supraspinatus tears, with 97.5% (n = 39) of affected patients. Subscapularis tears were found in 45.0% (n = 18) of patients and infraspinatus tears in 5.0% (n = 2). In terms of rotator cuff pathologies, MRI identified partial tears in 65.0% (n = 26) of the patients and complete tears in 35.0% (n = 14) (Table 1).

Comparison between USG and MRI

The sensitivity, specificity, PPV, NPV, and accuracy of USG in detecting partial and complete rotator cuff tears were compared with those of MRI as the reference standard. A comparison of USG and MRI findings showed that USG and MRI identified partial tears in 20 patients. However, a complete tear was identified in 13 patients. For partial tears, two patients were detected by USG who were not found on MRI. In addition, six patients with partial tears were detected by MRI but were missing by USG. Overall, 12 patients did not have partial tears on USG and MRI (Table 2).

**Table 2:** Comparison of USG and MRI for identification of partial tear

		MRI partial tear	
		Yes	No
USG partial tear	Yes	20	2
	No	6	12

In the evaluation of complete rotator cuff tears, USG demonstrated a high degree of concordance with MRI, which is the reference standard. Specifically, USG accurately identified 13 cases of complete tears confirmed by MRI (true positives). However, there were five instances where USG suggested the presence of a complete tear that MRI did not confirm (false positives). Conversely, MRI detected one complete tear that was missed by USG (false negative). Additionally, USG correctly identified 21 cases in which no complete tear was present, consistent with the MRI findings (true negatives) (Table 3).

**Table 3:** Comparison of USG and MRI for identification of complete tear

		MRI complete tear	
		Yes	No
USG complete tear	Yes	13	5
	No	1	21

USG demonstrated sensitivity, specificity, PPV, NPV, and overall accuracy of 76.92%, 85.71%, 90.91%, 66.67%, and 80.00%, respectively. MRI showed a sensitivity of 92.86%, specificity of 80.77%, PPV of 72.22%, NPV of 95.45%, and an overall accuracy of 85.00% (Table 4).

**Table 4:** Sensitivity and specificity analysis NPV, negative predictive value; PPV, positive predictive value

Identification method	Sensitivity	Specificity	PPV	NPV	Accuracy
USG	76.92%	85.71%	90.91%	66.67%	80.00%
MRI	92.86%	80.77%	72.22%	95.45%	85.00%

## Discussion

Rotator cuff tears are often observed in patients with shoulder pain. To evaluate rotator cuff pathologies, noninvasive imaging modalities, such as USG and MRI, have been employed in several studies [8,10]. USG can be used as a primary imaging modality because its accuracy in detecting both partial- and full-thickness rotator cuff tears is comparable to that of MRI, as reported in the literature [8,10,11].

Our study assessed the diagnostic performance of USG compared with MRI for detecting rotator cuff tears in a cohort of 40 patients. The cohort had a mean age of 46.12 ± 15.31 years, with a predominance of males (67.5%) over females (32.5%). Comorbid conditions such as diabetes mellitus and hypertension were observed in 35% and 22.5% of the patients, respectively. Singh et al. reported an age group of 56-65 years, including 36% of all participants. In addition, 56% of the participants were male [10]. Mehta et al. included 40 patients for the cross-sectional study, which also reported a male predominance where 26 were men and 14 were women. The age range reported was 41-50 years of age (24%) [12]. Bashir et al. also reported higher male predominance (56%) in the overall population. This study also reported an age range of 41-50 years [13].

The analysis revealed that the right shoulder was predominantly affected, with 82.5% of cases involving this side, whereas the left shoulder was affected in only 17.5% of cases. In terms of rotator cuff pathologies, USG identified supraspinatus tears in 82.5% of the patients, subscapularis tears in 40%, and infraspinatus tears in 5%. The extent of pathology detected by USG included partial tears in 55% and complete tears in 45% of the patients. In contrast, MRI demonstrated a higher prevalence of supraspinatus tears (97.5%) and partial and complete tears in 65% and 35% of patients, respectively. This aligns with the observations of Bouaziz et al., who reported a higher prevalence of right shoulder involvement (68%) than of left shoulder involvement (32%). In their study, most patients had supraspinatus tears (86.7%), with fewer cases of subscapularis (6.7%) and infraspinatus (3.3%) tears [14]. Similarly, Vijayvargiya et al. identified the supraspinatus tendon as the most frequently affected tendon in 90% of the cases [15]. Zlatkin also found that supraspinatus tendon involvement was predominant among their patients [16]. These findings were corroborated by the results of the present study.

A comparative analysis between USG and MRI highlights several key findings. USG showed a sensitivity of 76.92%, specificity of 85.71%, PPV of 90.91%, NPV of 66.67%, and overall accuracy of 80.00%. MRI showed a sensitivity of 92.86%, specificity of 80.77%, PPV of 72.22%, NPV of 95.45%, and an overall accuracy of 85.00%. MRI was used as the reference standard, and the comparison revealed that USG identified 20 out of 26 partial tears detected by MRI, with six partial tears missed by USG. Additionally, 12 patients showed no partial tear in either modality. USG accurately identified 13 complete tears confirmed by MRI and missed only one complete tear (false negative), while incorrectly diagnosing five cases as complete tears where MRI did not find any (false positives). This is because ultrasound is based on operator-dependent and obese patients, and restricted movement due to pain may interfere with detecting the exact findings. Notably, USG correctly identified 21 cases in which no complete tear was present (true negative). Singh et al. also reported similar findings, where MRI diagnosed the rotator cuff better in 40 patients along with 12 partial tears and 28 patients with full-thickness tears [10].

Mehta et al. reported that ultrasound demonstrated a sensitivity of 92.86% and a specificity of 100% for partial-thickness tears. For full-thickness tears, the sensitivity was 100%, while the specificity was 96.3% [12]. Comparable findings were observed by McMonagle and Vinson, Nunna et al., and Fischer et al., who demonstrated that both USG and MRI offer similar diagnostic capabilities, with ultrasound proving advantageous in cases requiring revision. Their study reported an accuracy of 91.1% for identifying tears in the supraspinatus tendon, 84.4% in the infraspinatus tendon, and 77.8% in the subscapularis tendon [5,17,18].

These findings underscore the overall effectiveness of USG in detecting rotator cuff tears, with notable alignment with the MRI results. The high sensitivity for complete tears and the relatively good sensitivity for partial tears suggest that USG is a reliable diagnostic tool. However, the lower sensitivity for partial tears compared with MRI indicates that while USG is effective, it may not always capture all cases of partial tears. The real-time imaging capability and cost-effectiveness of USG make it a valuable modality in clinical settings, complementing MRI for comprehensive shoulder assessment.

The results of this study support the use of USG as an accessible and effective tool for the initial evaluation of rotator cuff injuries, while MRI remains the superior modality for detailed assessment and confirmation, particularly for partial tears and comprehensive structural analysis.

Limitations

The study’s limitations include a small sample size, demographic bias toward males, a single-center design, operator dependency on ultrasound accuracy, a focus on rotator cuff injuries without considering other pathologies, and a lack of comparison with other imaging techniques. These factors may impact the generalizability and robustness of the findings, highlighting areas for improvement in future research.

## Conclusions

Our study showed that USG is a useful diagnostic tool for identifying rotator cuff tears. It has high sensitivity and specificity, making it an effective option for identifying rotator cuff pathologies. While USG is a cost-effective and real-time imaging method, MRI remains the gold standard for detailed assessment, particularly for partial tears and intricate structural evaluation. However, the high level of concordance between USG and MRI supports the use of USG as the primary diagnostic modality in clinical practice. Operator dependence, the build of the patient, and restricted movement due to pain may sometimes interfere with detecting the findings. It is important to note that MRI provides superior sensitivity and accuracy for comprehensive diagnosis and confirmation, particularly for partial rotator cuff tears. Therefore, integrating both imaging techniques can enhance diagnostic precision and inform effective management strategies for rotator cuff injuries.
